# A deep dive into fat: Investigating blubber lipidomic fingerprint of killer whales and humpback whales in northern Norway

**DOI:** 10.1002/ece3.7523

**Published:** 2021-05-01

**Authors:** Pierre Bories, Audun H. Rikardsen, Pim Leonards, Aaron T. Fisk, Sabrina Tartu, Emma F. Vogel, Jenny Bytingsvik, Pierre Blévin

**Affiliations:** ^1^ Akvaplan‐niva AS Fram Centre Tromsø Norway; ^2^ Department of Arctic and Marine Biology UiT ‐ The Arctic University of Norway Tromsø Norway; ^3^ Department of Environment and Health Vrije Universiteit Amsterdam The Netherlands; ^4^ School of the Environment University of Windsor Windsor ON Canada; ^5^ Centre d'Etudes Biologiques de Chizé Villiers en Bois France

**Keywords:** capital breeder, cetacean, herring eater, income breeder, LION/web, lipid, seal eater, stable isotope

## Abstract

In cetaceans, blubber is the primary and largest lipid body reservoir. Our current understanding about lipid stores and uses in cetaceans is still limited, and most studies only focused on a single narrow snapshot of the lipidome. We documented an extended lipidomic fingerprint in two cetacean species present in northern Norway during wintertime. We were able to detect 817 molecular lipid species in blubber of killer whales (*Orcinus orca*) and humpback whales (*Megaptera novaeangliae*). The profiles were largely dominated by triradylglycerols in both species and, to a lesser extent, by other constituents including glycerophosphocholines, phosphosphingolipids, glycerophosphoethanolamines, and diradylglycerols. Through a unique combination of traditional statistical approaches, together with a novel bioinformatic tool (LION/web), we showed contrasting fingerprint composition between species. The higher content of triradylglycerols in humpback whales is necessary to fuel their upcoming half a year fasting and energy‐demanding migration between feeding and breeding grounds. In adipocytes, we assume that the intense feeding rate of humpback whales prior to migration translates into an important accumulation of triacylglycerol content in lipid droplets. Upstream, the endoplasmic reticulum is operating at full capacity to supply acute lipid storage, consistent with the reported enrichment of glycerophosphocholines in humpback whales, major components of the endoplasmic reticulum. There was also an enrichment of membrane components, which translates into higher sphingolipid content in the lipidome of killer whales, potentially as a structural adaptation for their higher hydrodynamic performance. Finally, the presence of both lipid‐enriched and lipid‐depleted individuals within the killer whale population in Norway suggests dietary specialization, consistent with significant differences in δ^15^N and δ^13^C isotopic ratios in skin between the two groups, with higher values and a wider niche for the lipid‐enriched individuals. Results suggest the lipid‐depleted killer whales were herring specialists, while the lipid‐enriched individuals might feed on both herrings and seals.

## Graphical abstract

We documented an extended lipidomic fingerprint in two cetacean species. We were able to detect 817 molecular lipid species in blubber of killer whales and humpback whales. The profiles are described in terms of lipid classes. Through a unique combination of traditional statistical approaches and a novel bioinformatic tool, we showed contrasting fingerprint composition between species. The higher content of triradylglycerols in humpback whales is necessary to fuel their migration. The intense feeding rate of humpback whales prior to migration translates into an important accumulation of triacylglycerol content in lipid droplets. Upstream, the endoplasmic reticulum is operating at full capacity to supply acute lipid storage. There was also an enrichment of membrane components, which translates into higher sphingolipid content in the lipidome of killer whales, potentially as a structural adaptation for their higher hydrodynamic performance. The presence of both lipid‐enriched and lipid‐depleted individuals within the killer whale population in Norway suggests dietary specialization, consistent with significant differences in δ^15^N and δ^13^C isotopic ratios in skin tissue. This suggests the lipid‐depleted killer whales were herring specialists, while the lipid‐enriched individuals might feed on both herrings and seals.

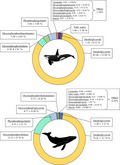

## 1 INTRODUCTION

To optimize their fitness, living organisms must acquire and allocate energy stores in a way that maximizes their survival and reproduction (Stearns, 1989). Adipose tissue is the primary and largest energy body reservoir where most lipids are stored, constituting the major source of metabolic fuel during periods of energy need (Irvine et al., 2017; Lafontan & Langin, 2009). In cetaceans, blubber is a specialized form of adipose tissue, densely packed with large spherical adipocytes, which can represent up to 50% of the total body mass in some species at certain life stages (Iverson & Koopman, 2018). Lipid composition of blubber consists mainly of triacylglycerols and reflect both diet and de novo lipogenesis (Bottino, 1978; Gross, 2020; Hansen & Cheah, 1969; Iverson & Koopman, 2018; Kawai et al., 1988; Krahn et al., 2001, 2004; Lafontan & Langin, 2009; Linder et al., 2010; Lockyer et al., 1984; Miller et al., 2020; Ruchonnet et al., 2006; Tilbury et al., 1997; Waugh et al., 2012). Blubber is also an endocrine and complex organ involved in a multitude of physiological and metabolic processes including immune response, energy balance, and lipid metabolism (Deyarmin et al., 2019, 2020; Galligan et al., 2018, 2020; Kershaw et al., 2018). Finally, blubber also serves as an efficient and adjustable thermal insulator, buoyancy adjuster, and hydrodynamic facilitator (Bagge et al., 2012; Iverson & Koopman, 2018; Lockyer, 1991). Consequently, investigating the lipid composition of blubber can provide key insights about the lifestyle of cetaceans including knowledge about dietary specialization, nutritional status, space‐use strategy, physiological state, and reproductive stage (Aguilar & Borrell, 1990; Bernier‐Graveline et al., 2021; Irvine et al., 2017; Mau, 2004.; Tang et al., 2018; Waugh et al., 2012; Linder et al., 2010; Ruchonnet et al., 2006).

Since 2011, several hundreds of killer whales (*Orcinus orca*) and humpback whales (*Megapetara noveangliae*) have gathered during the wintertime into some specific fjords of Troms in northern Norway to feed on the abundant schooling Norwegian spring‐spawning (NSS) herring (*Clupea harengus*) that for unknown reasons have started to overwinter in these areas (Jourdain et al., 2020; Rikardsen, 2019). Humpback whales are long‐range migratory species using the Norwegian Sea as a migration corridor. They travel between their summer feeding area characterized by high primary productivity mainly located in the northern Barents Sea east of the Svalbard archipelago (Leonard & Øien, 2020) and their breeding ground located in warm tropical waters of the West Indies or Cap Verde with low food availability during winter (Kettemer et al., in prep [https://whaletracking2018.uit.no/]; Stevick et al., ,^2003^, ^2006^). As capital breeders, humpback whales accumulate large energy reserves in the feeding grounds to meet the costs of growth, maintenance, locomotion, and future reproduction (Jönsson, 1997; Lockyer & Brown, 1981; Oftedal, 2011; Stephens et al., 2009; Waugh et al., 2012). By contrast, as income breeders, killer whales meet their energy demand throughout the year by continual foraging and thus have less need to store large energy reserves (Jönsson, 1997; Stephens et al., 2009). Killer whales that feed off northern Norway largely rely upon the NSS herring, following their yearly migration along the Norwegian coast (Dietz et al., 2020; Jourdain et al., 2020; Similä et al., 1996; Vogel et al., 2021). Nonetheless, diverse individuals' diet preferences including both fish and marine mammal prey and alternative space‐use strategy in the Barents Sea have also been indicated by stable isotopes and satellite tracking studies (Dietz et al., 2020; Foote et al., 2009; Jourdain et al., 2020).

Lipids function as a cellular energy reservoir and serve as major structural components of biological membranes such as plasma membranes and membranes of intracellular organelles (van Meer, 2005; van Meer & Vaz, 2005). They also participate in signaling as chemical messengers (Plakkal Ayyappan et al., 2016; Saliba et al., 2015). Glycerophospholipids and sphingolipids are major lipid constituents of cell membranes (Pomorski et al., 2001; van Meer et al., 2008), while triacylglycerols represent the bulk of lipid storage. The endoplasmic reticulum is a major biogenesis site of lipids, ultimately transported to other organelles as is the case for triacylglycerols in lipid droplets (Gregor & Hotamisligil, 2007; Lafontan & Langin, 2009; van Meer et al., 2008). Accordingly, lipid droplets can shrink and expand in a controlled manner in response to energetic demand and nutritional state (Kalantari et al., 2010). In short, when animals are feeding on a highly calorific diet, lipid storage increases and the lipid droplet expands in response to acute fat storage (Chitraju et al., 2012; Coelho et al., 2013).

To date, most studies investigating lipids in cetaceans only focused on a narrow snapshot of the lipidome, mainly on fatty acids and triacylglycerols (Bagge et al., 2012; Gross, 2020; Hansen & Cheah, 1969; Kawai et al., 1988; Krahn et al., 2004; Ruchonnet et al., 2006; Waugh et al., 2012, 2014). With the advent of state‐of‐the‐art analytical methods, together with the development of bioinformatics (e.g., Molenaar et al., 2019), the so‐called *"‐omics"* disciplines enable more comprehensive systemic profiling to understand the role of lipids in biological systems (Lydic & Goo, 2018; Miller et al., 2020). Thus, the objectives of this study were to document an extended lipidomic fingerprint in killer whales and humpback whales present in northern Norway during wintertime to better understand the determinants of lipid stores and uses in these cetaceans. (a) We described an extended lipid profile in blubber of killer whales and humpback whales. While triradylglycerols are expected to be the predominant lipid class in blubber for both species based on previous studies, we reasoned that the use of lipidomics in the present study would provide an in‐depth profiling and therefore likely enable the identification and quantification of other minor lipid classes in cetacean blubber, which have so far been overlooked (e.g., Bernier‐Graveline et al., 2021). (b) Through a unique combination of traditional statistical approaches, together with the use of a novel bioinformatic tool (Molenaar et al., 2019), we then compare the lipidomic fingerprint of both species. Although lipidomic composition similarities due to their phylogenetic affiliation were expected, the differences in response to their contrasted life‐history strategies were also anticipated. Specifically, we hypothesized higher proportion of lipid storage in humpback whales in order to cope with their ongoing long fasting and energy‐demanding migration at the time of sampling. (c) Finally, we investigated intraspecific variations of the lipidome within the killer whale population. Because of diverse individual dietary specialization within the killer whale population in Norway (Dietz et al., 2020; Jourdain et al., 2017, 2020), we sought to identify groups of individuals with closely related lipidomic fingerprint to search for specific feeding groups. To provide more information on potential dietary specialization in the killer whales, stable isotopes of nitrogen (δ^15^N) and carbon (δ^13^C) were determined in skin tissue of sampled individuals.

## 2 MATERIALS AND METHODS

### 2.1 Field sampling

Fieldwork was conducted in December 2017 (5 and 6) and January 2018 (13), in the coastal area of Skjervøy located in northern Norway (70°3′12.84″N, 20°57′23.07″E). We sampled 21 adult killer whales (18 males and 3 females) and 4 adult humpback whales (1 male and 3 females). All individuals were mostly biopsied on the main body in the region below or behind the dorsal fin with a biopsy dart with a 100‐mm hollow stainless‐steel tip (inner diameter: 6 mm, biopsy core maximum depth ~8.5 cm), shot from a crossbow. The dorsal region is enriched in lipids and considered as important fat storage, as compared to other body regions (Lockyer et al., 1984; Ruchonnet et al., 2006). The dart was attached to a string secured to the crossbow that enabled rapid recovery of the sample. Once collected, the biopsies were immediately removed from the dart to separate skin and blubber. Skin of humpback whales is generally thicker than skin of killer whales. The upper part of the skin (a few millimeters) was divided vertically into two parts, one part was used for stable isotope analyses (SIA) and the other was used for molecular sexing. The blubber closest to skin–blubber interface was used for lipid analysis (i.e., outer layer). Samples were snap‐frozen and stored in a dry shipper during field trip duration. Back at the field station, samples for lipid analysis and SIA were transferred directly into a −80°C freezer. The small piece of skin for molecular sexing was preserved in an ethanol‐filled 2‐ml cryotube at room temperature.

### 2.2 Molecular sexing

Sex of the whales was identified from skin samples by molecular sexing tests using the methods described in Bérubé and Palsbøll (1996). Each test consisted of one Y‐specific fragment and one X‐specific fragment in a multiplex PCR assay, as detailed in Appendix S1. Total genomic DNA was extracted in both killer whales and humpback whales using the DNeasy Blood & Tissue Kit (Qiagen), following the manufacturer's protocol (“Purification of total DNA from animal tissues” (spin‐column protocol)), as detailed in the SI).

### 2.3 Stable isotope analyses

Bulk nitrogen (δ^15^N) and carbon (δ^13^C) stable isotope ratios were analyzed from skin samples of killer whales (*n* = 19). The analyses were carried out following methods described in Marcoux et al. (2012) and Tartu et al. (2020). Briefly, skin samples were lyophilized at −48°C at a pressure of 133 × 103 mbar for 48 hr, homogenized into small pieces using a scalpel, lipid extracted using a 2:1 chloroform:methanol mixture to remove lipids (which can confound interpretation of δ^13^C data), and then the samples (400–600 μg) were placed into tin cups. Values of δ^13^C and δ^15^N were determined on a Delta V Advantage Thermo Scientific Continuous Flow Mass Spectrometer (Thermo Scientific, Bremen, Germany) coupled to a 4,010 Elemental Combustion System (Costech Instruments). Instrument accuracy met laboratory quality assurance criteria, as values were within 0.18‰ for NIST 8,547, NIST 8,573, and NIST 8,574 for δ^15^N, and 0.17‰ for NIST 8,573, 8,542, and 8,544 for δ^13^C. Precision was assessed with our laboratory standards (NIST 1577c, tilapia muscle, USGS 40, and Urea (*n* = 12 for each)), run every 15 samples, and was within 0.18. Bulk isotopes were used to investigate isotopic niche width as a proxy of the trophic niche (Newsome et al., 2007). The δ^13^C of a predator reflects the origin of food sources, indicating the sources of primary production in the food consumed by the whales, for example, pelagic versus benthic, inshore versus offshore (Hobson, 1999; Hobson et al., 1996; Kelly, 2000). Typically, inshore/benthic ecosystems are characterized by higher δ^13^C than pelagic/offshore ecosystems. The δ^15^N is commonly used as an indicator of the trophic position of a consumer (Hobson et al., 1994, 1996; Kelly, 2000; Newsome et al., 2007), owing to the stepwise enrichment from food source to consumer. Controlled diet experiment estimated the half‐life rates in skin of bottlenose dolphin (*Tursiops trucuntus*) to be 48 ± 19 days for nitrogen and 24 ± 8 days for carbon (Giménez et al., 2016). Therefore, isotopic values measured in killer whales are expected to represent the individuals' feeding habits in the 4–7 weeks prior sampling.

### 2.4 Lipidomic determination

#### 2.4.1 Lipid extraction from blubber

Blubber samples were weighed (killer whales: 164 ± 71 mg, humpback whales: 121 ± 35 mg) and added to Precellys tubes with ceramic beads. Homogenization of the sample was done during extraction of the lipids. 1 ml of ice‐cold (−20°C) acetonitrile/isopropanol (5:4, v/v) was added, and the samples were extracted with a Precellys 24 Dual device (Bertin Technologies) operated at 5196 rcf for two cycles of 10 s with a 15‐s break between the cycles. The Precellys cycle extraction program was repeated 15 times. 100 µl of Mili‐Q water was added, and the extract was centrifuged at 4°C for 10 min (7871 rcfG). The extract was then stored in glass vials at −80°C prior to lipidomic analysis. The extract was analyzed with an Agilent 1,290 Infinity UHPLC Binary Pump (G4220B) connected to a high‐resolution quadrupole time‐of‐flight mass spectrometer (MS, Compact QToF; Bruker Daltonics, Bremen, Germany). The HPLC system consisted of a pump, a vacuum degasser, an autosampler with a cooling unit (4°C), and a heated column compartment. The QToF was used in positive and negative modes to detect the lipids.

The MS parameter settings were defined as follows: capillary voltages of ±4500 V, end set plates of ±500 V, nebulizer gas (N_2_) pressure of 2 bar, drying gas flow rate of 6 L/min, and the drying gas temperature of 250°C. The m/z detection of MS was determined in the range from 50 to 2000 in positive mode with the sampling rate of 6 Hz in Auto MS/MS mode. Lipids were separated with a Kinetex EVO C18 column (100 × 2.1 mm, 2.6 µm particle size; Phenomenex, USA). The mobile phase consisted of A1 acetonitrile in water (60:40, v/v) with ammonium formate (10 mM) and formic acid (0.1%), and B1 isopropanol in acetonitrile (90:10, v/v) with ammonium formate (10 mM) and formic acid (0.1%). The gradient elution was as follows: 0 min 15% (B1); 0–2 min 30% (B1); 2–3 min 48% (B1); 3–20 min 82% (B1); 20–21 min 99% (B1); 21–30 min 99% (B1); and 30–32 min 15% (B1). Injection volume was 5 µl; the column temperature, 45 ℃; and the mobile phase flow rate, 0.25 ml/min.

#### 2.4.2 Data processing

Raw MS data were processed using Data Analysis (version 4.1; Bruker Daltonics). Internal mass calibration was performed with sodium formate injected in the first 40 s of each chromatographic run. Data were transferred to MZXL format and further processed with MS‐DIAL (Tsugawa et al., 2015) (v. 3.96) for annotation of the lipids, using the Lipidblast library (>35,000 MS/MS spectra) and following the shorthand nomenclature proposed by Liebisch et al. (2013) based upon the LIPID MAPS terminology (http://www.lipidmaps.org). The parameter settings were defined as follows: retention time begins at 0.5 min; retention time ends at 25 min; mass range begins at 60 Da; mass range ends at 2000 Da; MS1 (centroiding) tolerance at 0.005 Da; MS2 (centroiding) tolerance at 0.01 Da; smoothing level at 3 scans; minimum peak height at 500 amplitude; mass slice width at 0.05 Da; accurate mass tolerance (MS1) at 0.02 Da; accurate mass tolerance (MS2) at 0.02 Da; and identification score cutoff at 70%, without using retention information for scoring. Lipid concentrations were expressed in mg/kg wet weight (ww) based on lipid standards (Avanti Polar Lipids).

### 2.5 Statistics and data analysis

We first characterized the lipidomic fingerprint of blubber from killer whales and humpback whales. Molecular lipid species were grouped, and their concentrations summed according to which lipid class they belong to, in order to facilitate profile visualization. We then compared the composition of lipidomic fingerprint between killer whales and humpback whales (in terms of proportion, each lipid species represented in each sampled individual). Finally, we investigated intraspecific variations of lipidomic signature within the killer whale population. Proportions of each lipid species were normalized as the centered log‐ratio concentrations (ln(lipid_x_/∑lipid)) to minimize any potential misleading results (e.g., due to body size difference between species). Testing the influence of sex was not possible due to the imbalanced sex ratio in each species (humpback whales: 1 male and 3 females, killer whales: 18 males and 3 females). Except for analyses performed with LION/web (Molenaar et al., 2019), all statistics have been carried out using R version 4.0.3 (R core Team 2020). A significance level of *p*‐value = .05 was used for all tests.

#### 2.5.1 Interspecific comparison: killer whales versus humpback whales

We first performed a partial least‐squares discriminant analysis (PLS‐DA) on the lipidome using the *"mix.omics"* R package (Rohart et al., 2017) and tested for potential species clustering using the MANOVA Pillai's trace test from the *"car"* R package (Fox & Weisberg, 2019). The Pillai test is consistent with the use of an asymmetric data set. We further performed lipid ontology (LION) enrichment analysis to compare the lipidomic fingerprint of both species. To do so, we used a novel bioinformatic tool developed for global and in‐depth lipidomic data mining of mammalian system, the LION/web‐based interface (Molenaar et al., 2019). LION/web is an ontology database containing information related to lipid metabolism, which associates >50,000 lipid molecular species to biophysical, chemical, and cell biological features (referred as "LION terms"). In the present study, we used LION/web to connect the molecular lipid species with lipid classification, function, and distribution within the cellular components (i.e., the predominant cellular localization). As a general guideline, the lipid function branch comprises 3 subcategories: “membrane component” (i.e., associated with lipids that are primarily regarded as a structural component of lipid membranes), “lipid mediated signaling” (i.e., lipids that have been implicated in signaling), and “lipid storage” (i.e., lipids that are associated with storage). Enrichment analysis was performed using the ranking mode as described in Molenaar et al. (2019), on normalized lipid log‐ratio concentrations to compare the lipidomic fingerprint of killer whales and humpback whales and report any potential lipid LION term enrichments in the condition of interest. The analysis has been parameterized with killer whales as the control condition and humpback whales as the condition of interest. Calculated *p*‐values from LION/web were adjusted for multiple testing (i.e., Benjamini–Hochberg; Benjamini & Hochberg, 1995).

#### 2.5.2 Intraspecific variations in killer whales

We first performed a heat map to visualize the lipidomic fingerprint of each killer whale using the LION‐PCA heat map module of LION/web (Molenaar et al., 2019), which is a modified version from a gene ontology‐PCA (Wagner, 2015) specifically developed for global and in‐depth lipidomic data mining. Lipidomic signatures were sorted following a hierarchical clustering per individual in order to identify any potential groups of individuals with closely related lipidomic fingerprint. Differences between the two identified clusters (i.e., "lipid‐enriched" and "lipid‐depleted") were further confirmed with a PLS‐DA and the MANOVA Pillai's trace test. We also compared the nitrogen (δ^15^N) and carbon (δ^13^C) isotopic ratios of the "lipid‐enriched" and "lipid‐depleted" killer whales using linear models. The isotopic niche width (inferred from δ^13^C and δ^15^N) of the two identified groups was illustrated by standard ellipses (containing ∼95% of the data) on an isotopic biplot using the *“SIBER”* R package (Jackson et al., 2011). The areas of the resultant ellipses were then computed using both the maximum‐likelihood (SEAc, adjusted for small sample size) and the Bayesian approaches (SEAb; parametrized as detailed in Jackson et al., 2011). SEA is a measure of the standard deviation for bivariate data. Estimated SEA values were directly compared in a probabilistic manner in terms of similarity between the two groups of killer whales.

## 3 RESULTS

### 3.1 Lipidomic fingerprint in blubber of killer whales and humpback whales

**FIGURE 1:**
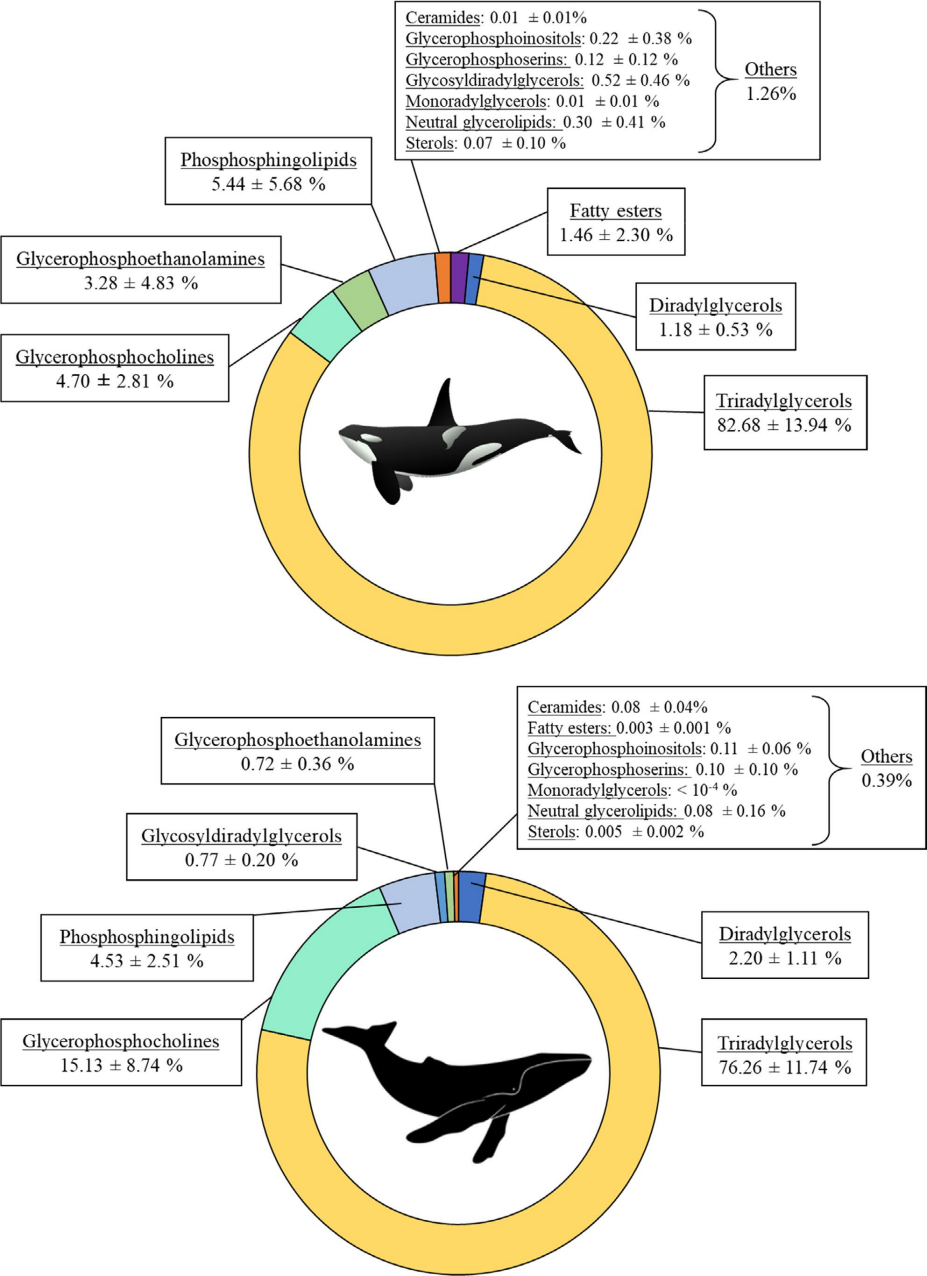
Proportion of lipid class (mean ± *SD*) in blubber lipidomic fingerprint of adult killer whales (*n* = 21) and humpback whales (*n* = 4) off northern Norway

In total, 817 lipid species were detected, clearly identified, and quantified in blubber of killer whales and humpback whales. The detected lipids were common to all samples, belonging to five lipid categories and 13 lipid classes (Table S1). Among the most predominant lipid classes were the triradylglycerols, the glycerophosphocholines, and the diradylglycerols with 325, 212, and 92 detected molecular lipid species, respectively (Figure 1 and Figure S1). Triradylglycerols constituted the main lipid class in both species, accounting for an average of 82.68% (range: 48%–97%) and 76.26% (range: 60%–87%) of the lipidomic fingerprint in blubber of killer whales and humpback whales, respectively (Figure 1). The lipidomic fingerprint of killer whales was then mainly composed of phosphosphingolipids (average 5.44%; range: 0.3%–19%), glycerophosphocholines (4.70%; 0.6%–10%), glycerophosphoethanolamines (3.28%, 0.06%–14%), fatty esters (1.46%; 0.03%–7%), and diradylglycerols (1.18%; 0.4%–2%) (Figure 1). By contrast, the lipidomic fingerprint of humpback whales was then mainly composed of glycerophosphocholines (15.13%; 8%–28%), phosphosphingolipids (4.53%; 3%–8%), and diradylglycerols (2.20%; 1.5%–4%) (Figure 1). In both species, each of the other lipid classes accounted for less than 1% of the lipidome.

### 3.2 Interspecific comparison: killer whales versus humpback whales

**FIGURE 2:**
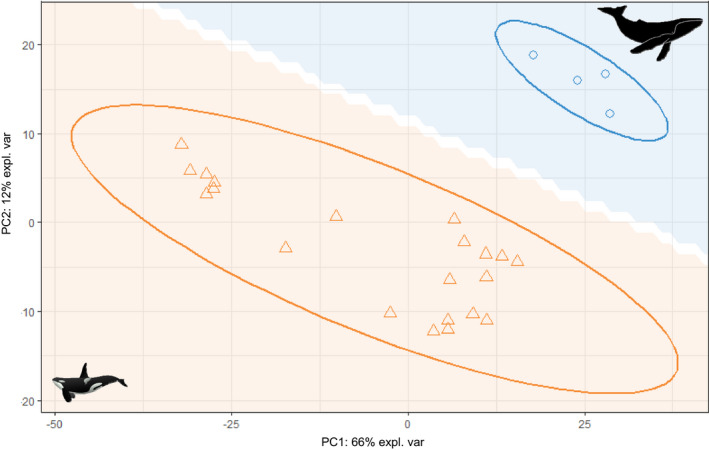
PLS‐DA of blubber lipidomic fingerprint in adult killer whales (*n* = 21; orange triangles) and humpback whales (*n* = 4; blue circles) off northern Norway. Ellipses represent 95% confidence interval. The colored background surface represents the prediction areas

**FIGURE 3:**
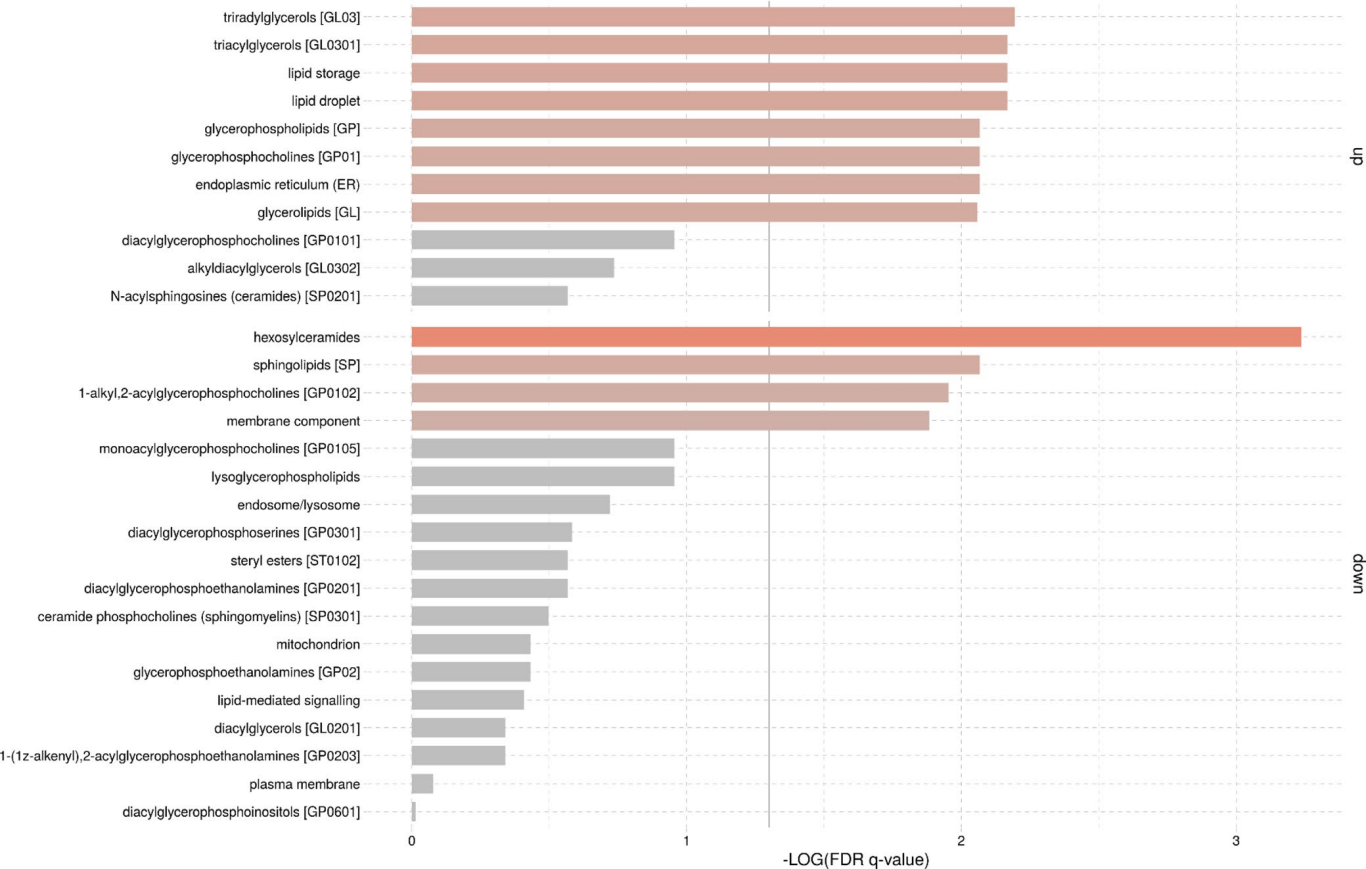
LION enrichment analysis of blubber lipidomic fingerprint composition in adult killer whales (*n* = 21) versus humpback whales (*n* = 4) off northern Norway. The gray vertical line indicates the cutoff value of significant enrichments. The size and color (from gray to red) of the horizontal bars (*x*‐axis) are scaled with the enrichment. Killer whales were considered as the control condition and humpback whales as the condition of interest. "up" refers to an enrichment, and "down" refers to a depletion

PLS‐DA indicated that blubber lipidomic fingerprint significantly differ between both species (Figure 2; MANOVA, Pillai's trace test, *F* = 20.98, *p*‐value < .001). Out of the 817 molecular lipid species, 791 were matched to the LION/web database (i.e., 97%), meaning that 26 lipid species were not considered in the LION/web analysis. This includes acylcarnitine (*n* = 1; lipid class: fatty ester), monogalactosyldiacylglycerol (*n* = 10; glycosyldiradylglycerols), and acylhexosylceramides (*n* = 15; neutral glycosphingolipids). Our results indicated significant enrichments in humpback whales (compared with killer whales) of the following LION terms: "triradylglycerols" (associated with *n* = 325 lipid species), "triacylglycerols" (*n* = 317), "lipid storage" (*n* = 329), "lipid droplet" (*n* = 329), "glycerophospholipids" (*n* = 272), "glycerophosphocholines" (*n* = 212), "endoplasmic reticulum" (*n* = 268), and "glycerolipids" (*n* = 417) (Figure 3). By contrast, our results indicated significant depletions in humpback whales (compared with killer whales) of the following LION terms: "hexosylceramides" (*n* = 17), "sphingolipids" (*n* = 75), "1‐alkyl, 2‐acylglycerophosphocholines" (*n* = 63), and "membrane component" (*n* = 434) (Figure 3).

### 3.3 Intraspecific variations in killer whales

**FIGURE 4:**
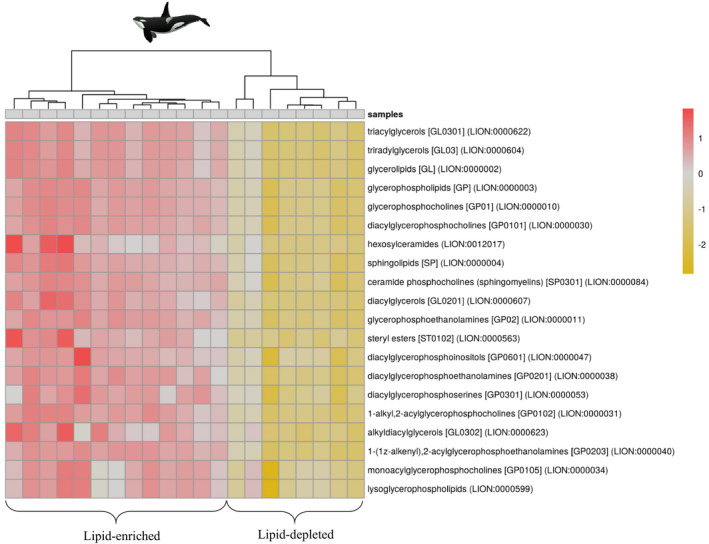
LION‐PCA heat map of blubber lipidomic fingerprints in adult killer whales (*n* = 21) off northern Norway. Lipids were sorted with a hierarchical clustering per individual (columns)

**FIGURE 5:**
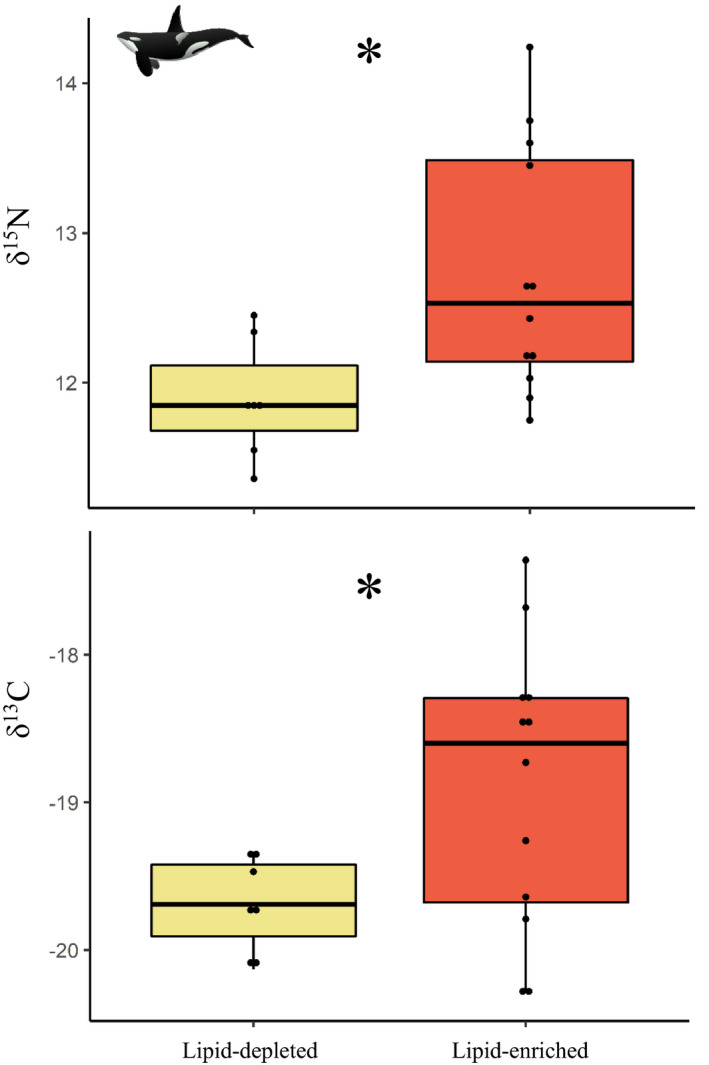
Comparison between δ^15^N and δ^13^C isotopic signatures between the "lipid‐enriched" (n = 12, red) and "lipid‐depleted" (n = 7, yellow) clusters in killer whales off northern Norway. Significant differences between the two groups are highlighted with an "*"

**FIGURE 6:**
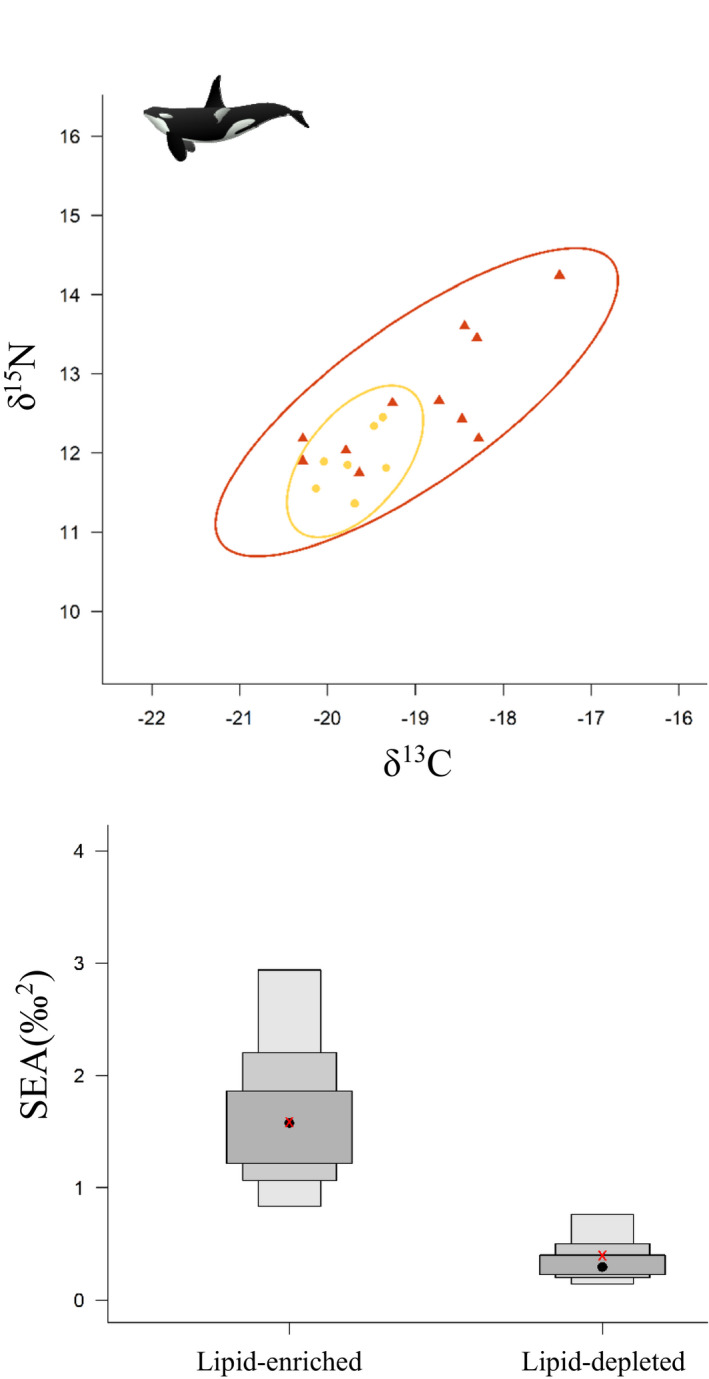
Isotopic niche width (inferred from δ^13^C and δ^15^N in skin) illustrated by standard ellipses (containing ∼95% of the data and computed with “SIBER” R package) of the "lipid‐enriched" (red triangles) and "lipid‐depleted" (yellow circles) killer whales off northern Norway (n = 19). Comparison of the standard ellipse area (SEA) between the two groups

The lipidomic fingerprints of killer whales appeared to cluster into two distinct groups (Figure 4). The first group is composed of 13 individuals with enriched lipid content (referred as "lipid‐enriched") compared with the second group composed of 8 individuals with depleted lipid content (referred as "lipid‐depleted"). The suggested difference in lipidomic fingerprint between the two groups was further confirmed by the PLS‐DA, which indicates significant differences (Figure S2; MANOVA, Pillai's trace test, *F* = 7.90, *p‐*value < .001). We also reported significant differences in δ^15^N (*F = *6.35; *p*‐value = .022; Figure 5), δ^13^C (*F* = 4.52; *p‐*value = .048; Figure 5), and isotopic niche width (probability = 1; Figure 6) between the two groups, with higher values and a wider niche in the lipid‐enriched killer whales.

## 4 DISCUSSION

To the best of our knowledge, this study provides the most comprehensive assessment of the lipidome in cetaceans. We were able to detect 817 molecular lipid species in blubber of killer whales and humpback whales. Through a unique combination of traditional statistical approaches, together with enrichment analyses using a novel bioinformatic tool specifically developed for global and in‐depth lipidomic data mining (Molenaar et al., 2019), we showed contrasting fingerprints between both species. Specifically, we found higher content of lipids involved in storage in humpback whales, which translates into higher triacylglycerol content stored in adipocyte lipid droplets. In contrast, killer whales showed higher content of membrane components, which translates into higher sphingolipid content. The reported differences in the lipidomic composition between both species are consistent with their contrasted life‐history strategy (i.e., capital versus income breeders). Finally, the presence of both lipid‐enriched and lipid‐depleted individuals within the killer whale population in Norway suggests dietary specialization, consistent with significant differences in nitrogen (δ^15^N) and carbon (δ^13^C) stable isotope ratios in skin tissue between the two groups.

### 4.1 Lipidomic fingerprint in blubber of killer whales and humpback whales

Triradylglycerols constituted the main lipid class in both species, accounting for an average of ~83% and ~76% of the lipidomic fingerprint in blubber of killer whales and humpback whales, respectively. The predominance of triradylglycerols was almost exclusively driven by high content of triacylglycerols and is consistent with previous investigations conducted on several cetacean species including harbor porpoises (*Phocoena phocoena*; Tilbury et al., 1997), gray whales (*Eschrichtius robustus*; Krahn et al., 2001), white whales (*Delphinapterus leucas*; Krahn et al., 2004), striped dolphins (*Stenella coeruleoalba*; Kawai et al., 1988), killer whales (Krahn et al., 2004), fin whale (*Balaenoptera physalus*; Lockyer et al., 1984; Ruchonnet et al., 2006), sei whale (*Balaenoptera borealis;* Bottino, 1978), and humpback whales (Waugh et al., 2012). Besides, these former studies largely focused on triacylglycerols, and so far, an exceedingly limited number of studies have focused on minor constituents, despite their essential functions (Bernier‐Graveline et al., 2021). With the advent of state‐of‐the‐art analytical methods, lipidomics enable a better systemic profiling. Accordingly, our results underscored the substantial contribution of glycerophosphocholines (~15%) and phosphosphingolipids (~5%) in the blubber lipidomic fingerprint of humpback whale, while pioneering studies conducted on other baleen whale species, the gray whale (Krahn et al., 2001), the sei whale (Bottino, 1978), and the fin whale (Lockyer et al., 1984), reported significantly lower phospholipid content (<4%, 3%, and ~5%, respectively). Similarly, our study highlights the substantial contribution of phosphosphingolipids (~5%), glycerophosphocholines (~5%), and glycerophosphoethanolamines (~3%) in the blubber lipidomic fingerprint of killer whale, while a former study conducted on the same species and on white whales failed to detect the presence of any phospholipids (Krahn et al., 2004). Therefore, the present study provides new knowledge and baseline information for future investigations on lipids and energy stores in marine mammals. Lipid concentrations reported in Table S1 should be interpreted with caution as lipid loss may have occurred at sampling due to the force of the biopsy cutting tip (Ryan et al., 2013). As a result, the lipid concentrations of blubber measured from biopsy darting might have been underestimated as compared to the tissue in situ. Such lipid loss could occur without affecting the lipid class profiles (Krahn et al., 2004; Waugh et al., 2014).

### 4.2 Interspecific comparison: Killer whales versus humpback whales

Blubber lipidomic fingerprint differs between killer whales and humpback whales. Yet, one might expect a higher degree of similarities between two phylogenetically affiliated species. Recently, a large‐scale analysis showed that changes in the mammalian lipidomes do not accumulate proportionally to the phylogenetic distances, but rather represent phenotypic differences including species‐specific adaptations (Khrameeva et al., 2018). In that respect, the reported difference in the lipidomic fingerprint composition between killer whales and humpback whales is consistent with their contrasted life‐history strategy.

Among the cetaceans, baleen whales are typically capital breeders, while toothed whales are generally income breeders (Irvine et al., 2017; Oftedal, 1997). Consistent with our results, Irvine et al., (2017) showed differences in cetacean energy stores between capital and income breeders in Australian waters, reporting that humpback whales accumulate more lipids than sperm whales (*Physeter macrocephalus*) in order to fuel their annual migration. According to Bengtson Nash et al., (2013), the utilization of humpback whale blubber lipids during the migration journey across the Southern Ocean was evidenced by an average of 23% reduction in lipids in outer blubber layer between early and late migrating cohorts. As capital breeders, humpback whales require to accumulate and store large energy reserves in their feeding grounds, in order to meet the costs of growth, maintenance, locomotion, and reproduction in the breeding grounds (Jönsson, 1997; Oftedal, 2011; Stephens et al., 2009; Waugh et al., 2012). Specifically, the humpback whales investigated in the present study belong to a long‐range migratory population using the Norwegian Sea as a migration corridor between their main summer feeding areas characterized with high primary productivity located in the northern Barents Sea east of the Svalbard archipelago and their breeding ground in warm tropical waters of the West Indies or Cap Verde with low food availability during late winter and spring (Kettemer et al., in prep [https://whaletracking2018.uit.no/; Stevick et al., 2003; 2006). The presence of humpback whales in northern Norway during wintertime represents a stop‐over area to refuel at an early stage of their migration route, and thus a unique opportunity to replenish their energy stores before undertaking a 6‐month fasting and energy‐demanding journey across the north Atlantic Ocean diagonal (Rikardsen, 2019). In adipocytes, we assume that the intense feeding rate of humpback whales translates into an important accumulation of triacylglycerol content in lipid droplets. Upstream, the endoplasmic reticulum is certainly operating at full capacity to supply the acute storage of lipids (Gregor & Hotamisligil, 2007; van Meer et al., 2008), which is consistent with the reported enrichment of glycerophosphocholines in humpback whales, major components of the endoplasmic reticulum (van Meer & de Kroon, 2011). To gain a complete understanding of lipid storage and shed light on mobilization of lipid species (Waugh et al., 2012), further studies investigating lipidomic fingerprint of humpback whales when they return from the migration are required. As income breeders, killer whales meet their energy demand throughout the year by continuous foraging, and thus, storing large reserves of energy is less essential (Jönsson, 1997; Stephens et al., 2009). Specifically, the killer whales investigated in the present study largely rely upon the NSS herrings (Jourdain et al., 2020; Mul et al., 2020; Similä et al., 1996; Vogel et al., 2021), following their yearly migration along the Norwegian coast (Dietz et al., 2020; Similä et al., 1996), although alternative space‐use strategy into the Barents Sea region toward Novaya Zemlya Island has been recently pointed out in three satellite‐tagged individuals (Dietz et al., 2020). As compared to the long‐range migration of humpback whales, killer whales travel across shorter distances and spend most of their time performing area‐restricted search behavior (Dietz et al., 2020; Mul et al., 2020; Vogel et al., 2021), indicating continual foraging and thus less lipid storage requirements.

In addition, the blubber has an important structural role and function as a streamlining tissue in cetaceans (Fish et al., 2008; Iverson & Koopman, 2018). Killer whales are efficient predators with specialized and elaborated feeding strategies, which require strong agility, high‐speed swimming, precise body movements, and maneuverability. In northern Norway, they are known to perform carousel feeding, which consists of herding the herrings into a tight and dense fish ball close to the surface, tail slapping the edge of the school, and eating stunned fish one by one (Similä & Ugarte, 1993). During the winter feeding on herring in the north Norwegian fjords, many of the killer whales are also documented to feed around the herring fishing vessels (Mul et al., 2020; Rikardsen, 2019). Moreover, previous field observations also reported killer whales chasing, harassing, and consuming harbor (*Phoca vitulina*) and gray (*Halichoerus grypus*) seals in Norwegian coastal waters (Jourdain et al., 2017; Vongraven & Bisther, 2014). In comparison, humpback whales are low‐speed swimmers taking benefit from their large body size and using lunge feeding to swallow large amounts of prey (Goldbogen et al., 2008; Simon et al., 2012). In northern Norway, humpback whales are known to perform surface lunging through herring balls herded by killer whales (Jourdain et al., 2017; Rikardsen, 2019). Hydrodynamic performances are thus expected to be higher in killer whales as compared to humpback whales. The present study reported an enrichment of membrane components and sphingolipid content in the lipidome of killer whales. Importantly, sphingolipids are known to harden membranes (Alonso & Goñi, 2018; van Meer & de Kroon, 2011) and reduce blubber compressibility (Ramstedt & Slotte, 2002). Then, the higher hydrodynamic performance of killer whales, as compared to humpback whales, might translate into stiffer membranes of adipocytes due to higher content of sphingolipids (i.e., hexosylceramides), as a swimming adaptation to minimize drag.

Although the present study provides relevant insights about lipids stores and uses in cetaceans, the low sample size of humpback whales precludes to make any firm conclusions and further studies including a larger dataset are needed. The use of remote biopsy darting to collect blubber and skin has marked a step forward to investigate physiological questions in free‐living cetaceans (Hunt et al., 2013). However, inherent challenges of this sampling technique do not allow systematic collection of the full blubber core. Thus, the findings presented here from the outer layer would gain benefits from complementary investigations conducted on healthy stranded animals, focusing on the full blubber core or the inner layer, which appears to be the most metabolically active blubber part (e.g., Lockyer et al., 1984; but see Waugh et al., 2014 and Aguilar & Borrell, 1990).

### 4.3 Intraspecific variations in killer whales

The presence of lipid‐enriched and lipid‐depleted individuals within the killer whale population in Norway suggests dietary preferences including prey species with contrasted nutritional value. Accordingly, Jourdain et al. (2020) investigated the dietary habits of killer whales in Norway based on δ^13^C and δ^15^N in skin tissue, together with concurrent visual field observations, and revealed individual dietary specialization. Their study underlined the occurrence of both fish specialist killer whales, reflective of local peaks in prey abundance, as well as seal eaters with a diverse diet composed of both fish and mammal prey throughout the year (Jourdain et al., 2020). Killer whales typically occur in large seasonal aggregations during the wintertime in northern Norway to feed on the abundant schooling NSS herring overwintering in these areas (Dietz et al., 2020; Jourdain et al., 2017; Rikardsen, 2019; Similä et al., 1996). Moreover, the presence of gray seals pupping grounds in northern Norway between September and December might represent a valuable pinniped food source at this time of the year (Haug et al., 1994; Nilssen, 2007). In that context, the lipid‐depleted killer whales might fall into herring specialists (38% of all individuals), while the lipid‐enriched individuals might feed on both herrings and seals (62% of all individuals).

Importantly, our conclusion about the diets of the two killer whale groups was supported by significantly higher values of δ^15^N and δ^13^C in lipid‐enriched killer whales as compared to the lipid‐depleted individuals, indicative of prey from higher trophic position and living in near‐shore coastal habitats. Moreover, the wider isotopic niche of the lipid‐enriched group is consistent with a mixed diet composed of both seals and herring, while the narrow and overlapping isotopic niche of the lipid‐depleted killer whales is indicative of prey specialization exclusively composed of herring (Foote et al., 2012; Jourdain et al., 2020; Samarra et al., 2017; Vogel et al., 2021). As a matter of evidence, average isotopic values measured in our study for each group (lipid‐enriched: δ^15^N = 12.7 ± 0.8‰ and δ^13^C = −18.9 ± 1.0‰; lipid‐depleted: δ^15^N = 11.9 ± 0.4‰ and δ^13^C = −19.7 ± 0.3‰) were perfectly in line with those reported in Jourdain et al. (2020) (seal eaters: δ^15^N = 12.6 ± 0.3‰ and δ^13^C = −18.9 ± 0.6‰; herring eaters: δ^15^N = 11.7 ± 0.2‰ and δ^13^C = −19.3 ± 0.3‰). With a relatively high nutritional value (Beck et al., 2003), the occurrence of seals in the diet of killer whales may be considered, at first glance, as being beneficial. However, this also raises the issue of exposure to environmental pollutants as *"preying on seals pushes killer whales from Norway above pollution effects thresholds"* (Andvik et al., 2020).

## CONFLICT OF INTEREST

None declared.

## AUTHOR CONTRIBUTIONS

**Pierre Bories:** Data curation (equal); formal analysis (equal); investigation (equal); writing–original draft (equal). **Audun H. Rikardsen:** Data curation (equal); methodology (equal); resources (equal); writing–review and editing (equal). **Pim Leonards:** Data curation (equal); methodology (equal); validation (equal); writing–review and editing (equal). **Aaron T. Fisk:** Data curation (equal); methodology (equal); validation (equal); writing–review and editing (equal). **Sabrina Tartu:** Writing–review and editing (equal). **Emma F. Vogel:** Writing–review and editing (equal). **Jenny Bytingsvik:** Conceptualization (equal); funding acquisition (lead); project administration (equal); supervision (equal); writing–review and editing (equal). **Pierre Blevin:** Conceptualization (equal); data curation (equal); formal analysis (equal); investigation (equal); project administration (equal); supervision (lead); validation (equal); writing–original draft (lead).

## Supporting information

Supplementary Material

## Data Availability

Data are available from the Dryad Digital Repository (https://doi.org/10.5061/dryad.cnp5hqc4d).
