# Supplementary material for: A deep dive into fat: Investigating blubber lipidomic fingerprint of killer whales and humpback whales in northern Norway
Source: Ecol Evol. 2021 May 1;11(11):6716–29. doi: 10.1002/ece3.7523 (PMC8207449; doi:10.1002/ece3.7523)

***Supporting information***

**A deep dive into fat: Investigating blubber lipidomics fingerprint of killer whales and humpback whales in northern Norway**

**Pierre Bories^1^, Audun H. Rikardsen^2^, Pim Leonards^3^, Aaron T. Fisk^4^, Sabrina Tartu^5^, Emma F. Vogel^2^, Jenny Bytingsvik^1^, Pierre Blévin^1*^**

^1^ Akvaplan-niva AS, Fram Centre, Tromsø, Norway

^2^ Department of Arctic and Marine Biology, UiT - The Arctic University of Norway, Tromsø, Norway

^3^ Department of Environment and Health, Vrije Universiteit, Amsterdam, The Netherlands

^4^ School of the Environment, University of Windsor, Windsor, Ontario, Canada

^5^ Centre d'Etudes Biologiques de Chizé, Villiers en Bois, France

***Corresponding author**

Pierre Blévin

Akvaplan-niva AS, Fram Centre

NO-9296 Tromsø, Norway

Email: [pbl@akvaplan.niva.no](mailto:pbl@akvaplan.niva.no)

**Molecular sexing**

Individuals were sexed using molecular sexing tests as detailed in Berube & Palsbøll (1996); one for Odontoceti (i.e., Killer whales) and one for Mysticeti (i.e., Humpback whales). Each test consisted of one Y-specific fragment and one X-specific fragment in a multiplex PCR assay yielding one band for females and two band for males. In the Odontoceti test, a male is characterized with the genotype 222/378, and a female with 378/378. In the Mysticeti test, a male is characterized with the genotype 209/241, and a female with 241/241. The PCR-reactions were carried out using 10 µl reaction volumes containing 1x Multiplex PCR Master Mix (Qiagen), 1 µl primer mix (ABI), 0.5 x BSA (NEB), 2.95 µl ddH2O (Qiagen) and 1 µl template DNA. PCR conditions were set up as follow: 10 min at 95°C, 32 cycles of 30s at 94°C, 30s at 58°C, 1 min at 72°C and final extension for 45 min at 72°C. Skin samples of white whales (*Delphinapterus leucas*) with known gender (2 females and 2 males) were included in the gender analysis as positive controls. One negative control was added every 7 sample analyses to ensure reliable results. The negative control included the PCR-reagents, with ddH2O instead of DNA. PCR products (1 µl) were mixed with Genescan 500 LIZ (Applied Biosystems) size standard (0.24 µl) and Hi-Di formamide (10 µl), following a 2 min denaturation at 95^o^C on a 2720 Thermal cycler. Capillary electrophoresis was carried-out on an ABI3730 DNA Analyzer (Applied Biosystems). The POP-7™ Polymer was used as a separation matrix and the sample injection time were set to 4 s/2kv. PCR fragments were analyzed in GeneMapper 4.1 (Applied Biosystems) and the peaks were manually checked.

**References**

Bérubé, M., Palsbøll, P. (1996). Identification of sex in Cetaceans by multiplexing with three ZFX and ZFY primers. *Molecular ecology* 5, 283-287

**Table S1:** Concentrations (expressed in mg.kg^-1^ ww) of lipids detected in blubber of adult killer whales (n = 21) and humpback whales (n=4) off northern Norway.

| Category | Class | Mean ± SD | | Number of molecular species |
| --- | --- | --- | --- | --- |
|  |  | **Killer whale** | **Humpback whale** |  |
| Fatty Acyl | Fatty esters | 88.28 ± 166.76 | 1.64 ± 0.85 | 1 |
| Glycerolipids | Monoradylglycerols | 4.01 ± 6.59 | 0.03 ± 0.02 | 1 |
|  | Diradylglycerols | 700.63 ± 989.24 | 1 471.6 ± 1201.77 | 92 |
|  | Triradylglycerols | 54 245.42 ± 59 339.41 | 46 534.17 ± 18 648.83 | 325 |
|  | Glycosyldiradylglycerols | 229.89 ± 249.48 | 510.78 ± 275.84 | 32 |
| Glycerophospholipids | Glycerophosphocholines | 1 815.35 ± 1 673.01 | 9 435.39 ± 6627.50 | 212 |
|  | Glycerophosphoethanolamines | 135.90 ± 96.44 | 458.51 ± 305.97 | 44 |
|  | Glycerophosphoinositols | 17.61 ± 15.23 | 65.65 ± 39.08 | 7 |
|  | Glycerophosphoserines | 29.75 ± 36.79 | 67.19 ± 65.47 | 9 |
| Sphingolipids | Neutral glycosphingolipids | 211.03 ± 288.67 | 53.68 ± 102.97 | 32 |
|  | Ceramides | 4.25 ± 4.50 | 55.5 ± 40.37 | 5 |
|  | Phosphosphingolipids | 1055.85 ± 888.95 | 2886.10 ± 1962.16 | 53 |
| Sterols | Sterols | 12.04 ± 23.06 | 2.87 ± 1.57 | 4 |

**
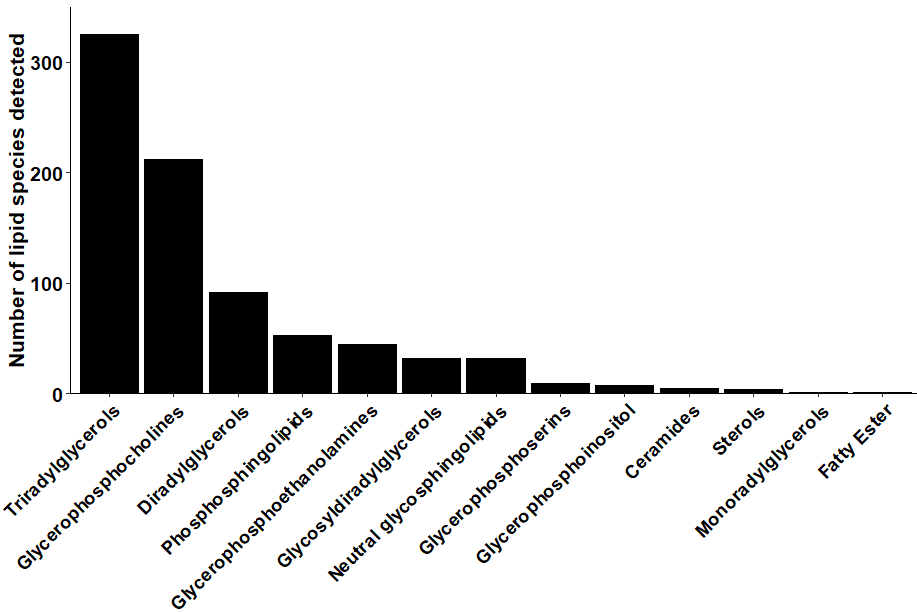
Figure S1.** Total number of molecular lipid species per lipid class detected in blubber of adult killer whales (n = 21) and humpback whales (n=4) off northern Norway.

**Figure S2.** PLS-DA of blubber lipidomics fingerprint in the "lipid-enriched" (n=13; orange triangles) and "lipid-depleted" (n=8; blue circles) killer whales off northern Norway. Ellipses represent 95% confident interval. The coloured background surface represents the prediction areas.


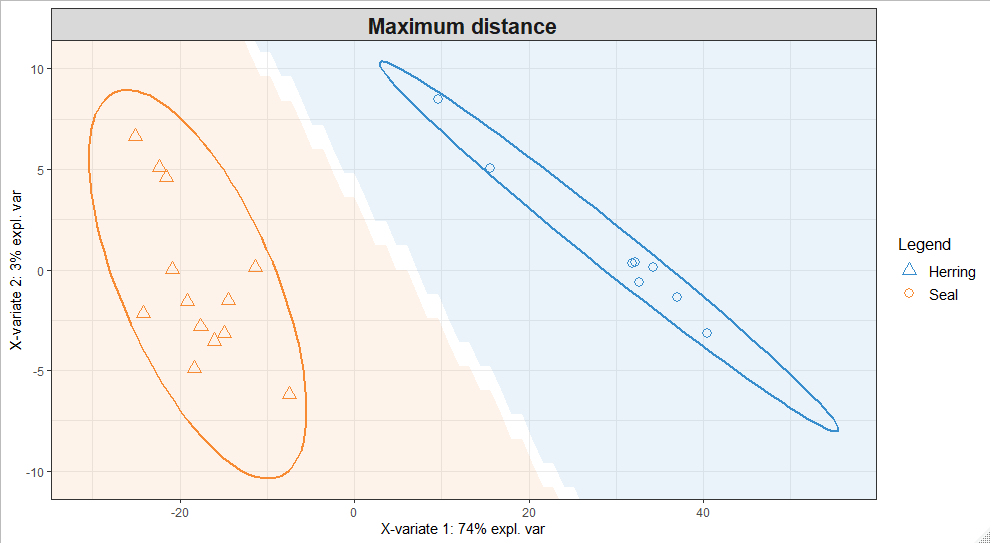


PC1: 74% expl. var

PC2: 3% expl. var


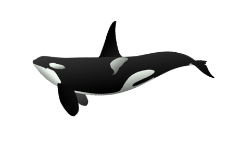

Supplement: Supplementary file 1 — Supplementary Material [file ECE3-11-6716-s001.docx]
